# Effect of non-pharmacological interventions on sleep in preterm infants in the neonatal intensive care unit: A protocol for systematic review and network meta-analysis

**DOI:** 10.1097/MD.0000000000027587

**Published:** 2021-10-29

**Authors:** Qingchun Huang, Xin Lai, Jianhua Liao, Yingchao Tan

**Affiliations:** a Department of Neonatology, The Central Hospital of Enshi Tujia and Miao Autonomous Prefecture, Enshi, Hubei Province, China; b Department of Pediatric Critical Illness, The Central Hospital of Enshi Tujia and Miao Autonomous Prefecture, Enshi, Hubei Province, China; c Department of Child Health Care, The Central Hospital of Enshi Tujia and Miao Autonomous Prefecture, Enshi, Hubei Province, China.

**Keywords:** neonatal intensive care unit, network meta-analysis, non-pharmacological interventions, preterm infants, protocol, sleep

## Abstract

**Background:**

Premature infants are prone to suffer multisystem complications after birth due to the incomplete development of organ tissues and low immunity, and they require a longer period of supervised treatment in the neonatal intensive care unit (NICU). However, due to the specificity of medical care in the NICU, the sleep of preterm infants is highly susceptible that has an impact on the prognosis of preterm infants. Recently, various non-pharmacological interventions have been applied to the sleep of preterm infants in the NICU, which have shown positive outcomes. However, the efficacy and safety of them are unclear. This study aims to evaluate the effects of non-pharmacological interventions on sleep in preterm infants in the NICU through a network meta-analysis.

**Methods:**

Randomized controlled trials of non-pharmacological interventions on sleep in preterm infants in the NICU published before September 2021 will be searched in online databases, including the Chinese Scientific Journal Database, China National Knowledge Infrastructure Database, Wanfang, China Biomedical Literature Database, PubMed, Cochrane Library, Embase, and Web of Science. Two researchers will be independently responsible for screening and selecting eligible literatures, extracting data and evaluating the risk of bias in the included studies. Stata 14.0 software will be used for data analysis.

**Results:**

The results of this meta-analysis will be submitted to a peer-reviewed journal for publication.

**Conclusion:**

This study will provide comprehensive and reliable evidence-based references for the efficacy and safety in different non-pharmacological interventions on sleep in preterm infants in the NICU.

## 1 Introduction

Globally, there are about 150,000 premature infants each year, ranking for the incidence of 1/10.^[1]^ Premature infants are prone to suffer multisystem complications after birth due to poorly developed organs and tissues and low immunity.^[2]^ Most preterm infants require an admission to the neonatal intensive care unit (NICU) for special medical care.^[3]^ However, excessive noise, prolonged high-intensity lighting, and frequent invasive procedures in the NICU significantly influence sleep in premature infants.^[4,5]^

Sleep is a very critical human physiological need, and preterm infants in the NICU are exposed to noxious stimuli that often disrupt and shorten their sleep duration.^[6]^ An adequate sleep is essential for neonatal growth and early neurosensory development. A growing number of evidences have shown that sleep quality is critical to brain development and synaptic plasticity, which is associated with long-term neurodevelopmental outcomes.^[7]^ It is reported that the NICU experience can overstimulate preterm infants, leading to sleep disruption, endocrine disruption, and even exacerbation.^[8,9]^ The risk of sleep-related sudden death in preterm, and low-birth-weight infants is 4 times higher than in the general population, and sleep-related sudden death accounts for 11.9% of total infant mortality.^[10]^ Therefore, sleep problems in preterm infants admitted in the NICU are a growing concern. How to protect and respect the sleep of preterm infants in the NICU, and how to properly assess and implement beneficial care measures have become important in the research of preterm infant development.

Both pharmacological and non-pharmacological interventions have been used to promote sleep quality. However, pharmacological interventions have potential adverse effects.^[11,12]^ Neonates are not recommended for the use of sedative and hypnotic drugs because they are in the rapid growth and neurodevelopment. Non-pharmacological interventions have been well highlighted in recent years,^[13,14]^ which have been used to improve sleep in premature infants in the NICU.

Previous studies have shown the effectiveness of various non-pharmacologic interventions for sleep in NICU preterm infants.^[15–21]^ However, evidence to determine their efficacy and safety of different non-pharmacological interventions is limited. Therefore, this study will perform a network meta-analysis on randomized controlled trials (RCTs) reporting the effects of non-pharmacological interventions on sleep in NICU preterm infants, thus providing a basis for clinical practice.

## 2 Methods

### 2.1 Study registration

This study has been registered in the OSF Registries (OSF registration number: DOI 10.17605/OSF.IO/NPR97), which follows the statement guidelines of preferred reporting items for systematic reviews and meta-analyses protocol.^[22]^

### 2.2 Eligibility criteria of inclusion of studies

#### 2.2.1 Types of studies

We will include all RCTs reporting the application of non-pharmacological interventions on sleep in preterm infants in the NICU.

#### 2.2.2 Types of participants

Premature infants admitted to NICU.

#### 2.2.3 Types of interventions

One or more non-pharmacological interventions, such as music therapy, touch, sound and light control, and sound and light control in experimental group. Conventional care measures or other types of interventions in control group.

#### 2.2.4 Types of outcome measures

1. Sleep efficiency;
2. Sleep time;
3.
Sleep behaviors.

### 2.3 Exclusion criteria

1. Duplicate publications;
2. Incomplete data;
3. Studies with inconsistent outcomes.

### 2.4 Data sources

RCTs of the application of non-pharmacological interventions on sleep in preterm infants in the NICU published before September 2021 will be searched in online databases, including Chinese Scientific Journal Database, China National Knowledge Infrastructure Database, Wanfang, China Biomedical Literature Database, PubMed, Cochrane Library, Embase, and Web of Science. MeSH terms and keywords will be used. In addition, citations of eligible literatures will be manually reviewed to avoid missing data.

### 2.5 Searching strategy

A combination of MeSH terms and free words will be adopted in the searching strategy. The detailed search strategy of PubMed was given in Table 1, and literature search in other online databases will be similarly conducted.

**Table 1:** Search strategy of the PubMed.

Number	Search terms
#1	Infant, Premature[MeSH]
#2	Neonatal Prematurity[Title/Abstract]
#3	Premature Infants[Title/Abstract]
#4	Preterm Infants[Title/Abstract]
#5	Infant, Preterm[Title/Abstract]
#6	Infants, Premature[Title/Abstract]
#7	Infants, Preterm[Title/Abstract]
#8	Premature Infant[Title/Abstract]
#9	Prematurity, Neonatal[Title/Abstract]
#10	Preterm Infant[Title/Abstract]
#11	OR/1-10
#12	Intensive Care Units, Neonatal[MeSH]
#13	Neonatal Intensive Care Units[Title/Abstract]
#14	Newborn Intensive Care Units[Title/Abstract]
#15	NICU[Title/Abstract]
#16	OR/12-15
#17	Sleep[MeSH]
#18	Sleep, Slow-Wave[Title/Abstract]
#19	Sleep, Slow Wave[Title/Abstract]
#20	Slow-Wave Sleep[Title/Abstract]
#21	OR/17-20
#22	Randomized Controlled Trials as Topic[MeSH]
#23	Clinical Trials, Randomized[Title/Abstract]
#24	Controlled Clinical Trials, Randomized[Title/Abstract]
#25	Trials, Randomized Clinical[Title/Abstract]
#26	Random∗[Title/Abstract]
#27	OR/22-26
#28	#11 AND #16 AND #21 AND #27

### 2.6 Data collection and analysis

#### 2.6.1 Literature screening and data extraction

**Figure 1:**
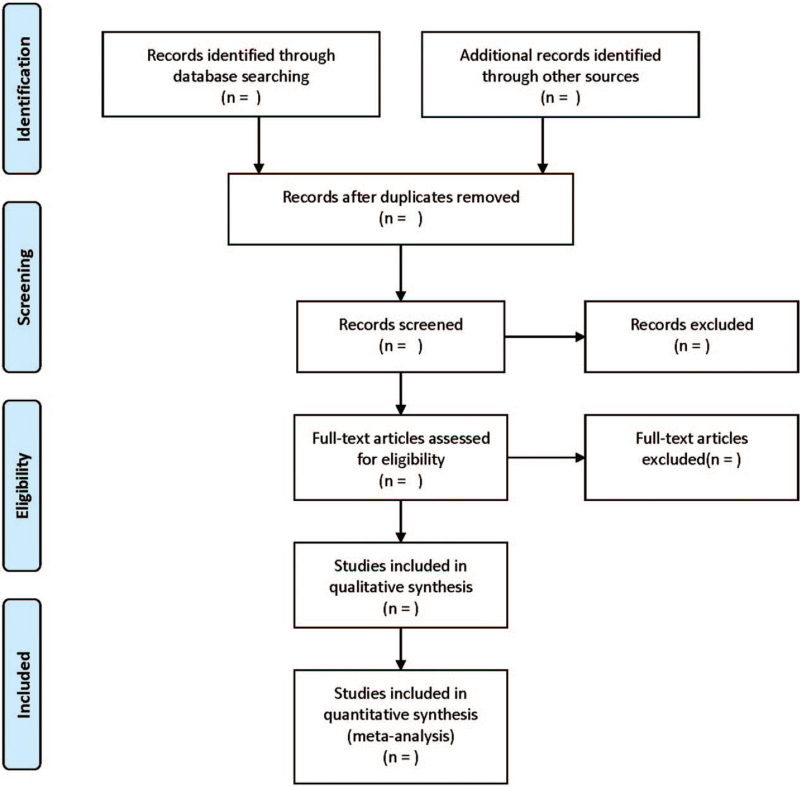
PRISMA flow diagram of the study selection process.

A Preferred Reporting Items for Systematic Reviews and Meta-analysis (PRISMA) flow diagram will be used to summarize the results of the whole selection process (Fig. 1). All studies retrieved from the database will be imported into the EndNote X8 software. After removing duplicates, 2 reviewers will independently screen the titles and abstracts of all studies. Full-text articles of potentially eligible studies will then be screened for further evaluation. Any discrepancies between the 2 reviewers will be resolved by a third reviewer. Data will be extracted independently by both reviewers using a standardized data extraction form. The following items will be extracted: First author, year of publication, country, sample size, age and sex of participants, intervention details, treatment duration and outcomes.

#### 2.6.2 Assessment of evidence quality

For each included study, methodological quality will be assessed independently by 2 reviewers using the Cochrane Collaboration's tool for assessing risk of bias in RCTs.^[23]^ The Cochrane risk of bias assessment includes 7 aspects, namely randomization methods, blinding of participants and investigators, blinding of evaluators, allocation concealment, outcome completeness, selective reporting of results, and other sources of bias. Bias will be assessed for each of the included RCTs as low risk of bias (low risk), high risk of bias (high risk), and uncertain (unclear).

#### 2.6.3 Measures of therapeutic efficacy

Continuous variables will be combined using standardized mean differences and 95% confidence intervals.

#### 2.6.4 Management of missing data

Missing data will be requested by Email; otherwise, the data will be excluded from the study.

### 2.7 Data synthesis

#### 2.7.1 Pairwise meta-analysis

Q-test with *I*^*2*^ test will be performed for assessing the heterogeneity of included studies. *I*^*2*^ < 50% and *P* > .10 will be considered as no statistical heterogeneity or less heterogeneity and a fixed-effects model will be adopted; otherwise, a random-effects model will be used.^[24]^

#### 2.7.2 Consistency check

The Wald test will be used to assess whether there is a difference between the direct and indirect evidence in the closed loop. *P* > .05 suggested a consistency between direct and indirect evidence within the closed loop, and a fitted consistency model will be used; Otherwise, an inconsistency model will be used and possible reasons for inconsistency will be explored further.

#### 2.7.3 Network meta-analysis

Network evidence plots will be depicted using the network plot command in Stata 14.0 software (STATA Corporation, College Station, TX). Network meta-analysis will be performed using the mvmeta command in Stata 14.0 software to combine direct and indirect comparisons and thus to produce a ranking between interventions. The surface under the cumulative ranking curves (SUCRA) will be used to rank the outcomes of the interventions, in which SUCRA is an indicator of the likelihood of an intervention being better or worse. SUCRA being closer to 100% indicates the better the efficacy of the intervention.

#### 2.7.4 Assessment of reporting biases

If the number of included studies for the outcome index is ≥10, the small sample effect and publication bias will be tested by drawing a comparison-correction funnel chart. The effect size of each indicator will be taken as the abscissa and the standard error will be used as the ordinate.^[25]^

#### 2.7.5 Subgroup analysis

Subgroup analyses will be performed based on the intervention time.

#### 2.7.6 Sensitivity analysis

The sensitivity analysis will be performed to test the stability of the results of meta-analysis.

#### 2.7.7 Ethics and dissemination

The content of this article did not involve moral approval or ethical review and would be presented in print or at relevant conferences.

## 3 Discussion

Sleep is important for maintaining the homeostasis of the organism.^[26,27]^ The sleep, growth development, recovery and prognosis of premature infants, whose systems are not yet fully developed, are often affected by their own complications, the disturbance of the NICU environment, and the stimulation of frequent pain.^[28–30]^ Non-pharmacologic interventions have been well concerned because of their low adverse effects. Although non-pharmacological interventions on sleep of premature infants in the NICU, measures and their outcomes are diverse. As a result, it is difficult to determine which non-pharmacological intervention is optimal in the efficacy and safety. This study aims to explore the effects of different non-pharmacological interventions on sleep in NICU preterm infants through a network meta-analysis, thus providing an evidence-based basis for making an optimal clinical decision.

Our study has several limitations. Due to the limitation of publication language, we will only include English and Chinese-published studies, which may lead to selective bias. In addition, the age, region, method of sleep assessment, and quality of literature may increase the likelihood of heterogeneity. We believe that our results will help identify the best interventions for decision-making, establish uniform and objective standards for sleep monitoring in preterm infants, conduct more high-quality RCTs with rigorous design, highlight the effects of non-pharmacological interventions on sleep in preterm infants and safety evaluation, and develop more clinically applicable interventions.

## Author contributions

**Conceptualization:** Yingchao Tan, Qingchun Huang.

**Data curation:** Xin Lai.

**Formal analysis:** Xin Lai.

**Funding acquisition:** Yingchao Tan.

**Investigation:** Xin Lai.

**Methodology:** Xin Lai, Jianhua Liao.

**Project administration:** Yingchao Tan.

**Resources:** Xin Lai, Jianhua Liao.

**Software:** Jianhua Liao.

**Supervision:** Yingchao Tan.

**Validation:** Jianhua Liao.

**Visualization:** Jianhua Liao.

**Writing – original draft:** Yingchao Tan, Qingchun Huang.

**Writing – review & editing:** Yingchao Tan, Qingchun Huang.
